# Beyond equity: Advocating theory-based health promotion in parallel with COVID-19 mass vaccination campaigns

**DOI:** 10.1016/j.puhip.2021.100142

**Published:** 2021-05-15

**Authors:** Ala'a B. Al-Tammemi, Zeinab Tarhini

**Affiliations:** a Department of Family and Occupational Medicine, Faculty of Medicine, University of Debrecen, H-4032, Debrecen, Hungary; b Doctoral School of Health Sciences, University of Debrecen, H-4032, Debrecen, Hungary; c Faculty of Medicine, University of Limoges, 87025, Limoges, France

**Keywords:** COVID-19 pandemic, COVID-19 vaccines, Vaccine hesitancy, Health promotion, Health behaviour

## Abstract

Despite the challenges in managing the COVID-19 pandemic waves in different contexts and capacities, the vaccines that were recently approved for use have created a window of hope to fight this pandemic more effectively by achieving herd immunity. However, the rates of vaccination coverage vary considerably between countries. While looking at COVID-19 vaccination from a different perspective, it brings up the following question: is equitable vaccine delivery and access the only critical issue? Assessing facilitators and barriers to successful vaccination initiatives should be carefully considered and addressed by subsequent actions. The COVID-19 vaccination campaigns as part of disease prevention programs could be embedded in the core of theories for a more systematic approach to enhance vaccine acceptance among people. For effective implementation of public health programs, it is imperative to understand human health behaviours and to have sufficient knowledge about cultural and environmental influences. Sufficient and satisfactory COVID-19 vaccine uptake is not only challenged by the availability of vaccines or their distribution, but also by cultural and social norms in the community as well as the complexity of human behaviours. Therefore, the global efforts should target communities with theory-based health promotion and awareness programs in parallel with vaccination campaigns as a part of public health practice.

## Highlights

• The COVID-19 pandemic is one of the most devastating events that impacted
various life domains globally.
• The recent development and approval of various COVID-19 vaccines has created
a window of hope to effectively combat the pandemic.
• Social, political, economic, and behavioural factors may play important roles in
COVID-19 vaccine uptake among people.
• Theory-based health promotion initiatives better be side by side with COVID-19
vaccination campaigns to enhance vaccine acceptance among people.

## 1 COVID-19 vaccines: the beacon of hope

The COVID-19 pandemic has caused many unprecedented changes in our life. In addition to afflicting people's physical and mental wellbeing, most life domains have been forced to change to meet the repercussions of the pandemic, including social life, economy, politics, health services delivery, and education [[1], [2], [3], [12]]. Despite all the challenges in managing the pandemic waves in different contexts and under various circumstances and capacities, the COVID-19 vaccines that were recently approved for use are the beacon of hope for millions of people to fight this pandemic more effectively through vaccine-accelerated herd immunity [11].

Globally, many countries have already started their vaccine rollouts under certain regulations and prioritization considering many factors such as age, comorbidities, and occupation. However, the distribution of COVID-19 vaccines and the rates of vaccination coverage vary considerably between countries [4]. On the other hand, recent large studies have reported different levels of willingness for vaccination and addressed the issue of vaccine hesitancy [[5], [6], [7], [8]]. Alongside the hesitancy issue, limited manufacturing capacity and global inequality in the distribution of COVID-19 vaccines were also labeled to be major concerns [8]. While looking at COVID-19 vaccination from a different perspective, it brings up the following question: is equitable vaccine delivery and access the only critical issue? Assessing facilitators and barriers to successful vaccination initiatives should be carefully considered and addressed by appropriate actions. The following sections will provide an insight into the previous question while looking at this issue from different perspectives.

## 2 Equity in COVID-19 vaccination: vaccine availability versus human health behaviours

Human behaviours are characterized by their dynamicity, complexity, and diversity [9]. For effective implementation of public health programs, for example, a vaccination campaign, it is imperative to understand the human health-related behaviours (e.g., how to make a decision that affects health) and to have sufficient knowledge about cultural and environmental influences. Changing health-related behaviour is very challenging and sophisticated, considering the inter-linked roots of religious, economic, and social influences [9]. Literature has addressed that successful health promotion campaigns better be grounded in various health behavioural theories. Thus, assessing facilitators and barriers to accept a healthy behaviour (e.g., to accept vaccination) should be carefully considered and addressed by subsequent actions.

Therefore, we stress the point that availability of and accessibility to COVID-19 vaccines is one of the challenges that many communities may face, however, the untold side of the story could be more challenging as it is embedded in a complex human behaviour that is affected by different factors across the world. A scenario of available vaccines but still low vaccine acceptance among people in certain contexts is on the table. Equitable distribution of vaccines should be accompanied by supportive messages using mass media campaigns to enhance vaccine acceptance, and as proposed by the World Health Organization “With a fast-moving pandemic, no one is safe, unless everyone is safe”. Using technology in health services delivery, education and socialization was a unique remark of the current pandemic. Hence, utilizing social media platforms and other accessible communication channels to deliver reliable and credible information to people about the efficacy of COVID-19 vaccines and their satisfactory safety levels is considered a cost-effective and time-efficient way to support vaccination campaigns. The effectiveness of such method could be affected by various cultural and social norms that should be appropriately targeted by the awareness campaigns as well.

## 3 Theory-based health promotion campaigns in parallel with COVID-19 vaccine rollouts

We propose that the COVID-19 mass vaccination campaigns as part of disease prevention programs could be embedded in the core of the *Structural Model of Health Behaviour* for a more systematic approach to enhance vaccine acceptance among people and to effectively tackle any obstacles in vaccine delivery chains. This model was developed by Cohen and colleagues in the 2000s [10] and the model has combined the inter-related factors that influence health behaviours at the population-level rather than individual-level. Therefore, the COVID-19 vaccination and its awareness campaigns should target the four constructs of this model, namely: *availability, physical structures, social structures, as well as cultural and media messages.*

By employing the first construct of the abovementioned structural model, availability, and easy accessibility to various types of COVID-19 vaccines can speed up the vaccination process and enhance vaccine uptake among people. Manufacturers and the COVAX alliance may consider timely production of a sufficient supply of various types of COVID-19 vaccines to meet the current requirements. This calls for collaborative efforts involving high-income countries to provide sufficient financial and logistic support for the vaccine alliance. It is worth mentioning that the concept of availability can be affected by the global and country-level policies as well. For example, in many countries, a certain type of COVID-19 vaccine is delivered to people while the people may prefer another type that is not available in the country or available with limitations.

The second construct of the model is the physical structure, which in the current vaccine case, refers to the actual characteristics of the vaccines themselves and the delivery facilities. This factor may enhance or reduce the vaccine uptake. Targeting people with information about the safety and efficacy of the developed vaccines from credible resources and preparing well-organized facilities to provide vaccines can encourage people to accept vaccines as a health behaviour of favourable outcomes. In this domain, it is also vital to maintain transparency of information and to effectively utilize digital health channels to organize and track the vaccination campaigns; thus, maintaining an unruffled process. The procedures of vaccine delivery in various settings should also consider the COVID-19 precautionary measures such as physical distancing and effective triaging system.

The third construct of the model is social structures which reflect the policies and regulations that influence certain health behaviour, and these structures could act directly without changing people's attitudes and beliefs or indirectly by changing social norms [10]. Respecting people's autonomy is crucial because mandatory vaccination could be against the ethical principle of respect for autonomy. However, the country's policies should consider the impacts of herd immunity when a considerable proportion of the community is vaccinated. As most people and countries were significantly impacted by the economic and social changes, easing up the economic, social and travel restrictions can be proposed to the community upon sufficient vaccination level and to those who got their vaccine jabs. In this way, people may become more willing to take vaccines into their consideration.

Lastly, utilizing cultural and mass media messages in an organized way is expected to help in raising awareness about the importance of the COVID-19 vaccines. Providing people with clear practical messages based on previous outbreaks and infectious diseases that were successfully eradicated or prevented by vaccines is exceptionally vital. However, these messages should consider the level of health literacy of the targeted community.

In summary, sufficient and satisfactory COVID-19 vaccination coverage is not only challenged by the availability of vaccines or their distribution, but also by cultural and social norms in the community as well as the complexity of human health behaviours. Addressing the factors raised in each construct of the described model is expected to help with tackling areas that slow down the progress of vaccination campaigns in a more systematic way. Therefore, the global efforts should target communities with theory-based health promotion and awareness programs side by side with the COVID-19 vaccination campaigns as a part of public health practice.

## Conflict of interest

The authors have no conflicts of interest to declare that are relevant to the content of this article.

## Funding

This article did not receive any specific grant from funding agencies in the public, commercial, or not-for-profit sectors.

## Ethical approval

Not required.
